# Hospital Libraries

**Published:** 1919-06-14

**Authors:** 


### HOSPITAL LIBRARIES.

### A Scheme for Centralisation.

APART from the main library, which most hospitals with medical schools possess, one finds that there are several departmental libraries scattered about the respective departments, such as those for physiology, bacteriology, chemistry, pharmacology, etc. As a rule they are only used by the officers in charge of these departments, and have no connection whatever with the main library—they are entirely on their own, and out in the cold as it were.

Now that hospitals are concentrating their efforts upon reconstructions, it would be a great advantage if these departmental libraries were to join hands and co-operate with the main hospital library to supply students and others with any book they may require. Such a scheme is already working successfully at one of the largest London hospitals.

Usually hospital libraries have a very restricted allowance for the purchase of books, binding, journals, etc.; consequently their means of purchase are very limited. The only way to meet the demand for books is by subscribing to a special medical circulating library, such as Lewis's, where practically every modern text-book can be obtained.

Departmental libraries can prove themselves far more useful than one imagines, as usually they contain a number of special books and monographs, not to be found in the main hospital library. In order that the student may derive full advantage from the department in which he is particularly interested, why not definitely associate and correlate these smaller libraries with the main library?

These libraries could be controlled quite easily by the librarian of the main library in the following manner.

1. By ensuring that all books in the main library and departmental libraries are catalogued on the card-index system. 2. By allocating coloured cards to departmental library books, or else stamping the name of the respective department on the same coloured card. 3. Access to departmental libraries be given to those holding a requisition signed by the librarian, stating the book he desires to consult. 4. That distinction should be made between books of reference on the one hand and text-books on the other. Students and others wishing to consult such text-books should obtain these volumes only in the main hospital library, and not have the right to requisition text-books in common use in the departmental libraries. 5. The exact method of making out and dealing with the main library cards referring to volumes in the departmental libraries should be left to the librarian. 6. Every department should have a catalogue of the books in their respective departmental libraries. 7. The method of arranging the books and shelves be left to the head of the department concerned. 8. The keys of the departmental book-cases should be kept by the head of the department or by one of his assistants.

As soon as these libraries are established, certain volumes, periodical or otherwise, which are little used in the main library but which could be referred to more frequently in the departmental library concerned, might with mutual advantage be transferred to such departments from the main library.

In order to ensure that departmental libraries remain intact and that no loss occurs as the result of borrowing books from time to time upon requisition from the main library, it is suggested that an annual report from the heads of each department possessing a library should be presented to the library committee.

